# Progressive 3D Printing Technology and Its Application in Medical Materials

**DOI:** 10.3389/fphar.2020.00122

**Published:** 2020-03-20

**Authors:** Daoyang Fan, Yan Li, Xing Wang, Tengjiao Zhu, Qi Wang, Hong Cai, Weishi Li, Yun Tian, Zhongjun Liu

**Affiliations:** ^1^ Department of Orthopedic, Peking University Third Hospital, Beijing, China; ^2^ Engineering Research Center of Bone and Joint Precision Medicine, Ministry of Education, Beijing, China; ^3^ Beijing National Laboratory for Molecular Sciences, State Key Laboratory of Polymer Physics & Chemistry, Institute of Chemistry, Chinese Academy of Sciences, Beijing, China; ^4^ University of Chinese Academy of Sciences, Beijing, China; ^5^ Department of Pediatrics, Peking University Third Hospital, Beijing, China

**Keywords:** additive manufacturing, 3D printing, functional biomaterials, tissue engineering, 3D pharmacological models, medical apparatus

## Abstract

Three-dimensional (3D) printing enables patient-specific anatomical level productions with high adjustability and resolution in microstructures. With cost-effective manufacturing for high productivity, 3D printing has become a leading healthcare and pharmaceutical manufacturing technology, which is suitable for variety of applications including tissue engineering models, anatomical models, pharmacological design and validation model, medical apparatus and instruments. Today, 3D printing is offering clinical available medical products and platforms suitable for emerging research fields, including tissue and organ printing. In this review, our goal is to discuss progressive 3D printing technology and its application in medical materials. The additive overview also provides manufacturing techniques and printable materials.

## Introduction

As an additive manufacturing (AM) technique, three-dimensional (3D) printing enables customized fabrication of 3D constructs based on computer aided design (CAD) software or images obtained from computed tomography (CT) and magnetic resonance imaging (MRI). Firstly developed in the 1980s, 3D printing technology was called rapid prototype technology and has been well applied in a variety of industries with different printing techniques and materials (Liaw and Guvendiren, 2017). With the rapid development of 3D printer, the overall 3D printing market grew to $9.9 billion in 2018 and is expected to reach $34.8 billion in 2024 (MarketsandMarkets, 2019). The medical 3D printing market is expected to maintain significant growth due to the huge potential demand for costumed medical products. Currently, with the expiry of many 3D printing patents (including stereolithography and selective laser sintering), 3D printers and products are becoming cheaper and easy to access (Rahman et al., 2018).

3D printing technology has been widely applied in a variety of industries including aviation (Wong, 2016), geoscience (Ishutov et al., 2018), education (Smith and Jones, 2018), clothing (Markstedt et al., 2017), medical (Mitsouras et al., 2015; Giannopoulos et al., 2016), and pharmaceuticals (Orsi et al., 2015; Norman et al., 2017; Trenfield et al., 2018). Among these medical and pharmaceutical industries, orthopedic and dental applications are favorable to embrace the 3D printing technology (MarketsandMarkets, 2019). It is related to the demand for the patient-specific design and fabrication of the final devices (such as joint prosthesis, surgical guides, and dental restorations) (Eltorai et al., 2015; Tahayeri et al., 2018). Personalized devices manufactured preoperationally are benefited for the efficiency and accuracy (Konta et al., 2017). For medical education and surgical planning, 3D anatomical models are printed subtly with microscopic anatomy structures (Mukherjee et al., 2017; Ganguli et al., 2018). Tissue and organ printing is an emerging field that mainly focused on regenerative medicine and tissue engineering by both academy and industry (Murphy and Atala, 2014). Based on it, preclinical patient-specific disease models are used for drug testings and screenings. 3D printing technology is merging with traditional pharmaceuticals for the development of dose-customized drugs (Norman et al., 2017).

**Figure 1:**
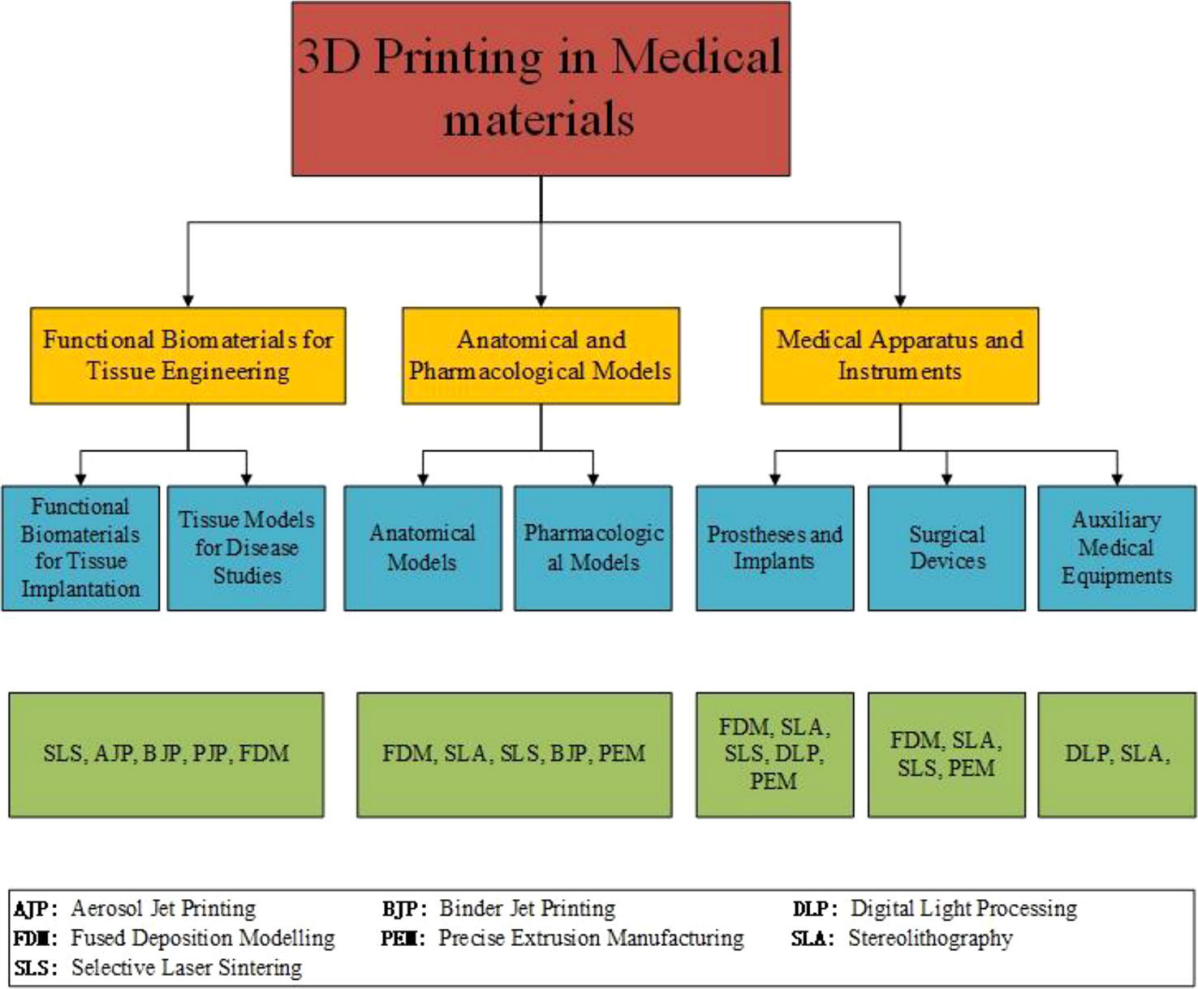
Progressive 3d printing technology and its application in medical materials. Chart showing the application area (yellow boxes) with corresponding products (blue boxes) and primary 3D printing techniques (green boxes).

In this review, recent techniques and applications of 3D printing in medical materials are well summarized. Common AM techniques and printable materials are presented for better understanding of their potential, limitations, and applications. Medical applications including tissue engineering, anatomical models, apparatus, and instruments with 3D printing technology are also provided and summarized. We finally demonstrate our concluding remarks and future outlook on 3D printing in medical materials (**Figure 1**).

## Current am Technologies and Printable Materials

There are about two dozen AM techniques, among which only some techniques are widely applied in medical industry. The main reason is the specific fabrication process and raw material to meet the high-quality requirements for medical devices. Four common AM techniques are powder-based printing (Brunello et al., 2016), vat polymerization-based printing (Stefaniak et al., 2019), droplet-based printing (Graham et al., 2017), and extrusion-based printing (Taylor et al., 2018).

### Powder-Based Printing

**Figure 2:**
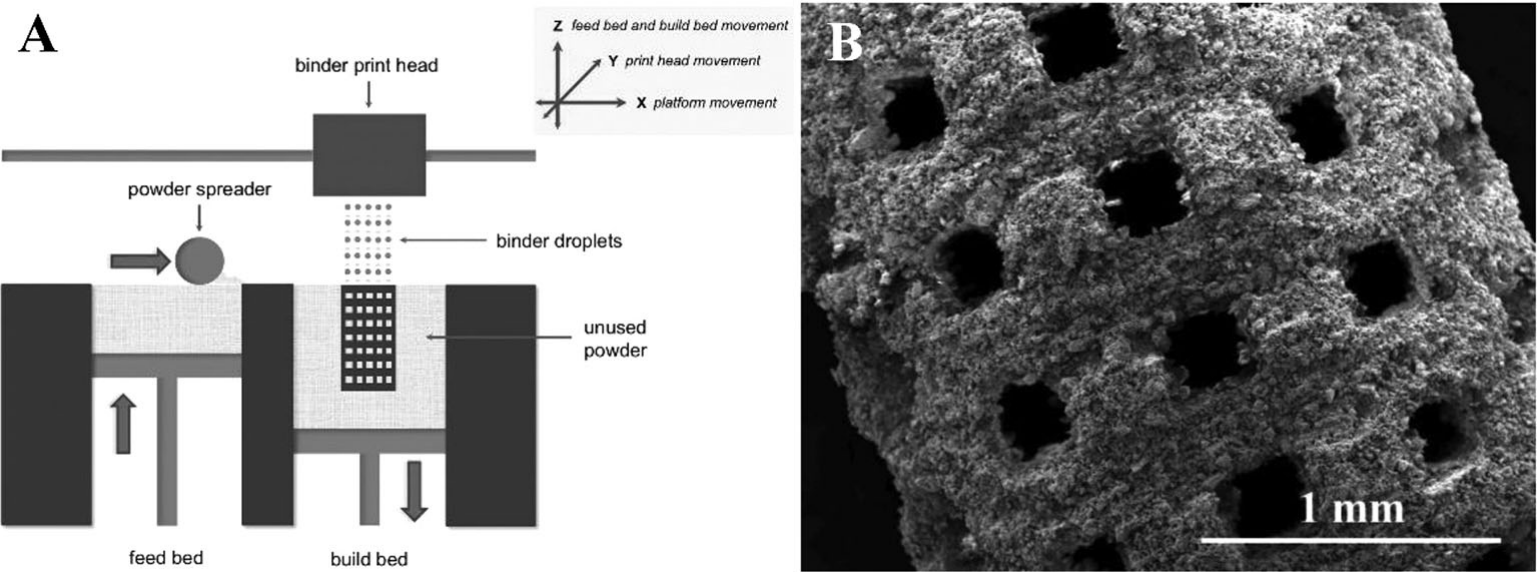
Printing process of powder-based printing and related products. **(A)** Schematic diagram. **(B)** Products manufactured by powder-based printing method. Reproduced, with permission, from (Brunello et al., 2016).

Powder-based 3D printing is a promising technique with excellent ability for customized fabrication with a variety of external shapes, internal structures, and porosities. There are four common powder-based printing techniques: selective laser sintering (SLS), selective laser melting (SLM), direct metal laser sintering (DMLS), and electron beam melting (EBM) (**Figure 2**) (Brunello et al., 2016). Every technique is based on localized heating to generate melted metallic powder, which would be used to fabricate the customized products. There are obvious differences in both printing process and product characters among these four powder-based printing techniques. For SLS and DMLS, powder particles are bounded with laser instead of spray solution. In the printing process, the laser draws specific patterns on one layer of the powder bed (Fina et al., 2017). The roller in the printer distributes a new layer of powder onto the surface once the printing of the previous layer is completed. After being built layer-by-layer, printed objects are collected underneath the powder bed. As a specific kind of SLS, DMLS utilizes metal exclusively. Different from sintering techniques, SLM and EBM fully melt powder with laser and electron beam respectively (Wysocki et al., 2018). For the work of electron beam, the powder bed in the EBM printer maintains high working temperature (> 870 K). It directly affects the quality of the fabrication especially in the details of microstructure. Comparatively, products printed with SLM maintain higher tribological, mechanical, and corrosion properties. With the differences between sintering and melting, the surfaces of products printed with sintering techniques (SLS and DMLS) are rough as powders are not completely melted.

Although the sintering techniques produce products with rough surfaces, they can process with a large variety of materials including plastic powder, ceramic powder, and metal alloys. As the high working temperature, material with volatile constituents (Mg, Zn, Bi, etc.) are not feasible for EBM, while SLM can treat a much wider spectrum of metallic alloys including Ti-based, Al-based, Fe-based, Ni-based, Cu-based, Co-based, and their composites. However, the melting process brings a big advantage that it can produce fully dense parts without post-treatment steps such as infiltration or thermal process.

### Vat Polymerization-Based Printing

**Figure 3:**
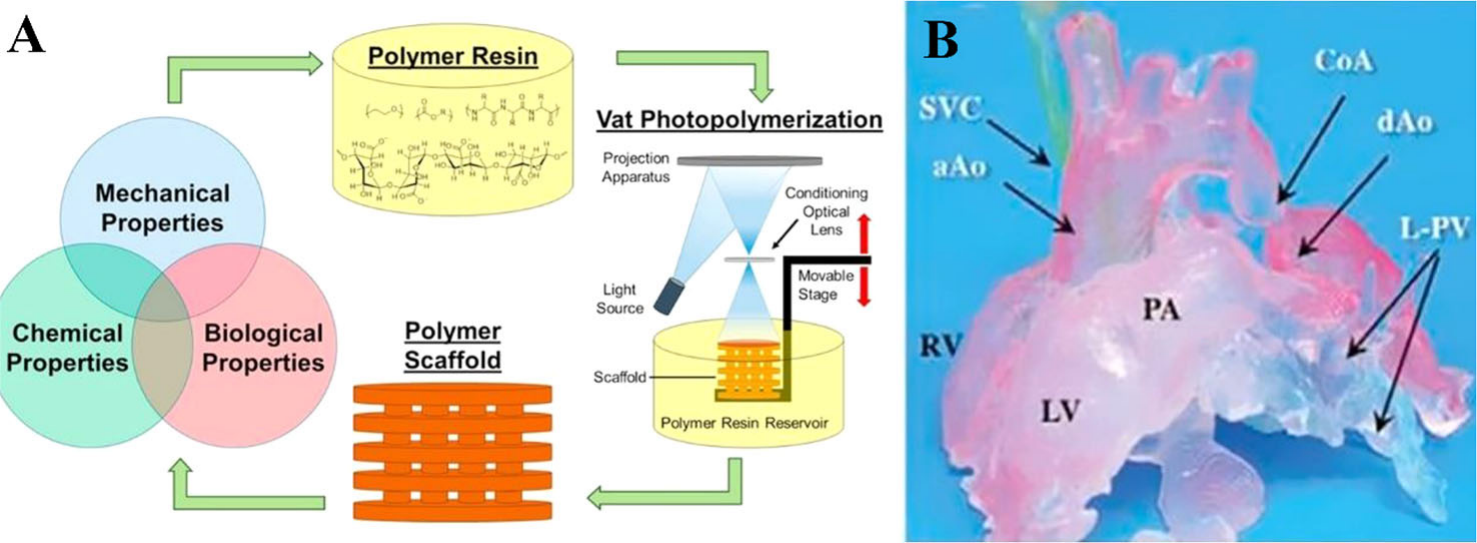
Polymer scaffold fabricated with SLA approach. **(A)** Schematic diagram. **(B)** Products manufactured by vat-polymerization based printing method. Reproduced, with permission, from (Mondschein et al., 2017).

Vat-polymerization based printing technique is based on light curing resin material and light selective hardening polymerization molding. It is widely used for fabricating complex devices with functional parts such as valves, lenses and fluidic interconnects (Carve and Wlodkowic, 2018). In the process, a vat of photosensitive polymer resin is selectively exposed to a specifically controlled beam of leaser or light (Credi et al., 2016; Credi et al., 2018). The polymer is polymerized after spatially localized irradiation to fabricate the specific constructions (Credi et al., 2016; Wang et al., 2018a). Common process includes digital light processing (DLP), stereolithography (SLA), and multiphoton polymerization (MPP) (**Figure 3**) (Carve and Wlodkowic, 2018). SLA was the first AM technology applied in medicine in 1994 (Dittmann et al., 1994). A spot laser irradiates the resin localized in a single x-y direction in SLA (Zanchetta et al., 2016), whereas a digital illuminant irradiates the whole x-y plan in DLP (Osman et al., 2017). For both SLA and DLP, the print platform moves parallelly to the z-axis while the final product is fabricated layer-by-layer (Zanchetta et al., 2016; Osman et al., 2017). Differently in MPP, the photosensitive polymer resin is irradiated by a femtosecond laser beam thoroughly in multi directions, resulting that it is not a layer-by-layer technology (Wollhofen et al., 2018). Products printed with vat polymerization technology need to be exposed to light after printing to enhance stability (Credi et al., 2016; Carve and Wlodkowic, 2018).

### Droplet-Based Printing

**Figure 4:**
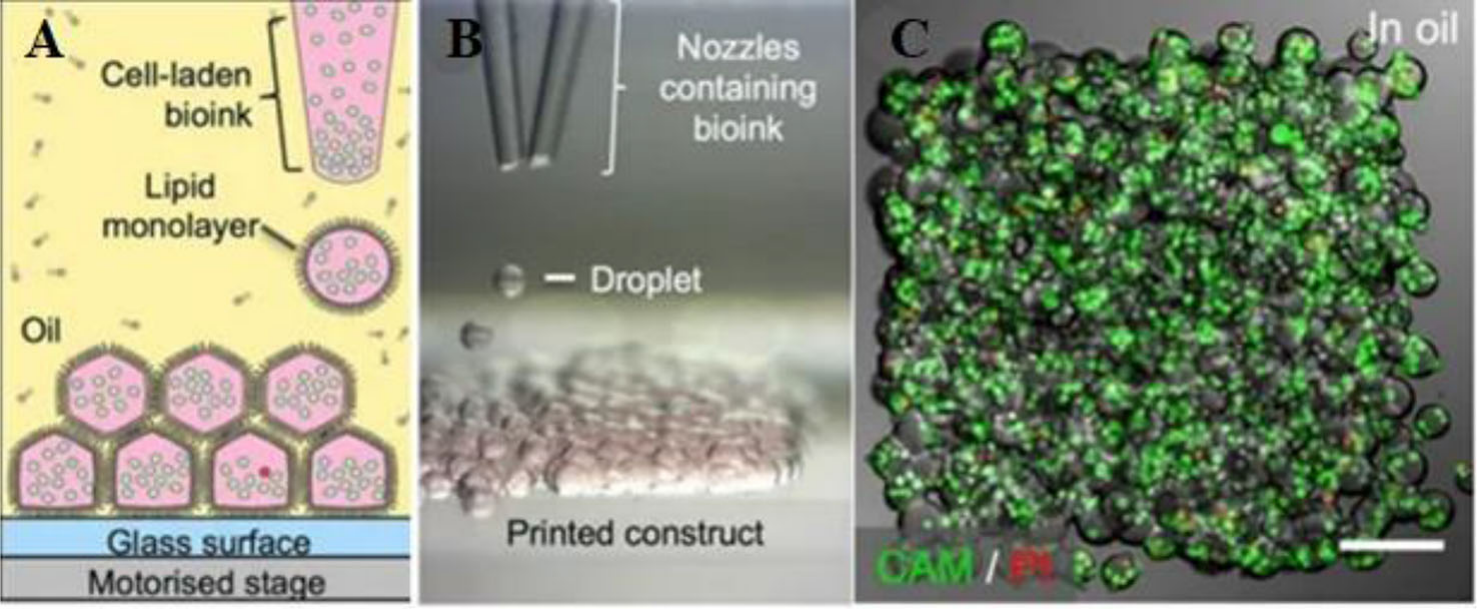
3D printing of droplet-based printing. **(A)** Schematic diagram of droplet-based cell printing. **(B)** Bright-field micrographs of patterned cell junctions containing two cell types. **(C)** Confocal fluorescence micrographs of cell constructs printed under oil. Reproduced, with permission, from (Brunello et al., 2016).

Material jetting technology is a process where droplets of liquid materials are ejected and polymerized throughout hundreds of jets. The polymerization only occurs selectively by directed UV for designed structures (Revilla-Leon and Ozcan, 2019). Material jetting technology includes aerosol jet printing (AJP), binder jet printing (BJP), and poly jet printing (PJP) (**Figure 4**). During AJP, composite in aerosol suspension droplets is carried *via* N_2_ gas and ejected onto the substrate layer by layer (Yuan et al., 2017). Multi materials including metals, polymers, and ceramics can be used in AJP with a low printing temperature, which is benefit for biomanufacturing (Mahajan et al., 2013). Binder jet printing (BJP) is similar with SLS except that BJP do not need thermoplastic excipient (Hong et al., 2016). The binder in BJP should meet specific ranges of surface tension (35–40 mJ/N) and viscosity (5–20 Pa**·**s) (Kim D. H. et al., 2018). In PJP, polymer resin drops are cured by UV light immediately without time consuming postprocessing (Revilla-Leon and Ozcan, 2019). With high resolution, PJP is capable of printing refined structures (Carve and Wlodkowic, 2018).

### Extrusion-Based Printing

**Figure 5:**
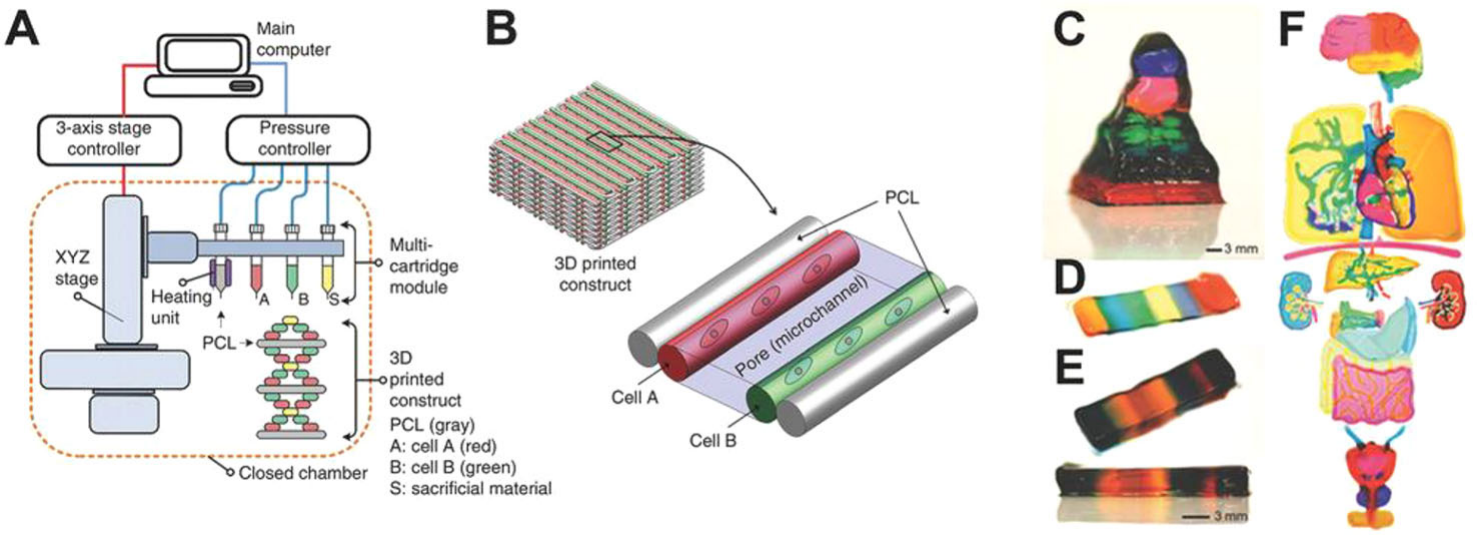
3D printing of extrusion-based multi-layer printing. **(A)** Schematic diagram of extrusion-based printing. **(B)** Multi materials printed with two cell types. **(C–F)** Available complex organs printed with extrusion-based printing techniques. Reproduced, with permission, from (Placone and Engler, 2018).

Extrusion-based printing was firstly developed by S. Scott Crump in 1988, commonly referred as fused deposition modeling (FDM) or fused filament fabrication (FFF) (Placone and Engler, 2018). FDM is a mature technology based on the extrusion of thermoplastic or composite materials drawn through the hot extrusion head (with one/multiple extrusion nozzles) (Paxton et al., 2017). Fused materials were deposited layer by layer with the horizontal and vertical movement of nozzles controlled by numerically-controlled machine tool (Ozbolat and Hospodiuk, 2016). Extrusion-based printing widely applied in metal printing, polymer printing, and bioprinting (**Figure 5**) (Ning and Chen, 2017). The printing techniques have been recently developed to precision extrusion deposition (PED) (Fedore et al., 2017), precise extrusion manufacturing (PEM) (Jamroz et al., 2018), and multiple heads deposition extrusion (MHDS) (Serex et al., 2018). Multiple bioprinting applications in vascular models, soft-tissue models, and bone models manufactured with extrusion-based printing technology have been well-developed in recent years (Ahlfeld et al., 2017; Paxton et al., 2017; Ahlfeld et al., 2018). One major advantage of its bioprinting application is that the hydrogels of extrusion-based printing is capable to fabricate products with high cell density (> 1 × 10^6^ cells ml^−1^) (Petta et al., 2018; Taylor et al., 2018; Chen et al., 2019).

## Applications in Medical Materials

AM technologies have been widely applied in medical materials, especially in tissue engineering, medical models, medical instruments, and drug formulations. A variety of printing technologies and products have lightened the broad market of medical and chemical applications of 3D printing.

### Functional Biomaterials for Tissue Engineering

**Figure 6:**
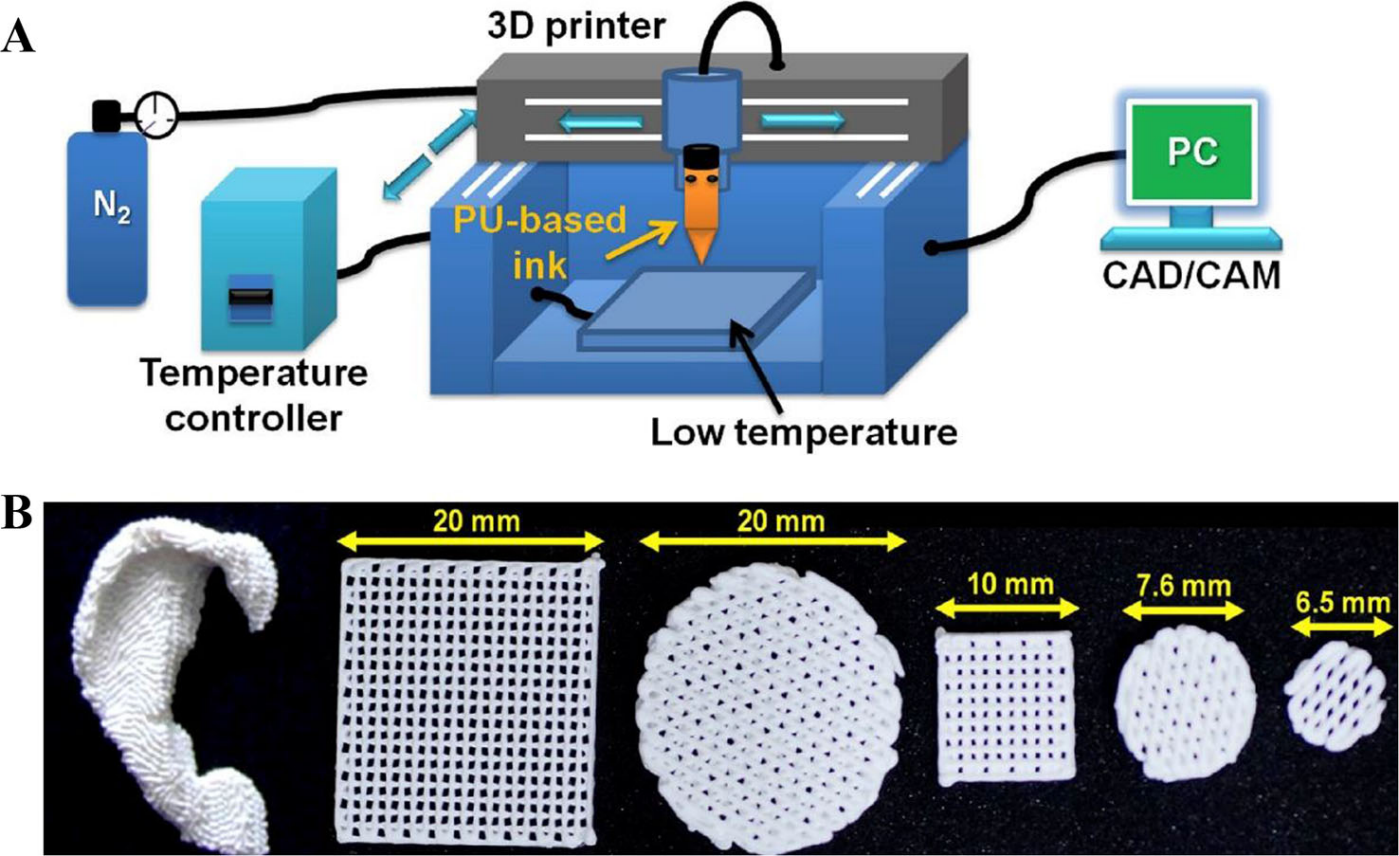
Functional biomaterials and related printing technique. **(A)** Schematic of a 3D printing platform for performing a water-based biological scaffold. **(B)** Appearance of 3D printed brackets in various shapes and sizes. Reproduced, with permission, from (Hung et al., 2016).

Tissue engineering with 3D printing has been focused on two parts, functional biomaterials for tissue implantation and tissue models for disease studies. In this section, functional biomaterials manufactured with AM technologies would be the focus. Tissue scaffolds are important component of 3D printing tissue engineering as they can provide structural supports for cell attachment, proliferation and migration (**Figure 6**). Tissue engineering scaffolds and basic medical scaffolds are considered different especially in biological activity and application purposes (Yang et al., 2018). Good bioactivity, excellent biocompatibility, and appropriate mechanical property are three basic requirements for an ideal tissue engineering scaffold. While basic medical scaffolds are usually applied for filling tissue coloboma or fixation without requirement for bioactivity. Implantable tissue engineering scaffolds are required to be degradable where scaffolds would be replaced by palingenetic tissues (Wang et al., 2018b). To induce tissue or bone growth inside the scaffolds, traditional procedures including molding, freeze drying, and electrospinning have been applied in the manufacture. However, none of the traditional procedures can fabricate scaffolds with customized mechanics, architecture and porosity. With the development of AM, scaffolds with high resolution, customized design, and high porosity have been successful in medical applications.

Tissue engineering scaffolds are fabricated in two major methods, printing with cells mixed in ink or gel and seeding cells onto scaffolds post printing. The most common methods applied in scaffolds fabrication are vat polymerization, SLS, BJ, and FDM. Inkjet printing and extrusion-based printing are the two popular bioprinting technologies, while bio-scaffolds are fabricated based on or without scaffolds. For bioprinting techniques based on scaffolds, hydrogels or polymers laden with cells are cured with AJP, BJP, PJP, and vat polymerization. For bioprinting techniques without scaffolds, hydrogels filled with high cell density (> 1 × 10^6^ cells ml^−1^) are applied directly relying on cell-cell interactions. Cells in such density need to fuse and mature in the bioreactor for a period of time.

Only a few companies have launched commercial tissue engineering scaffolds. Organogenesis Inc, one of the world's most famous FDA-approved 3D printed medical device supplier, introduced their GINTUIT™, a tissue engineering product approved in 2012 for oral soft tissue repair and regeneration. It is a commercialized cell and gene therapy product combined fibroblasts and keratinocytes in bovine collagen. In 2016, another famous supplier, Stryker released the product Tritanium^®^ LP, a titanium lumber posterior cage. The lumber cage is fabricated with abundant porous by DMLS technology with titanium alloy. The inner porous are helpful for blood vessel and bone growth inside the lumber cage.

With widespread concerns from various industries, bioprinting and tissue engineering have made significant progress and wide applications. Applications covered profuse tissues including tooth, bone, cartilage, ear, blood vessel, liver, kidney, and myocardium (Zhu et al., 2019). In 2017, Monica M. Laronda et al. from Northwestern university claimed successful fabrication of a bioprosthetic ovary created using 3D printed microporous scaffolds restoring ovarian function in sterilized mice (Laronda et al., 2017). Recently, Byoung Soo Kim and his colleges developed human skin with PJP 3D printing system (Ahn et al., 2016). This printed skin showed favorable biological characteristics, including stable dermis and epidermal layers. Manufactured skin substitutes can significantly improve skin healing of the wound area.

### Anatomical and Pharmacological Models

**Figure 7:**
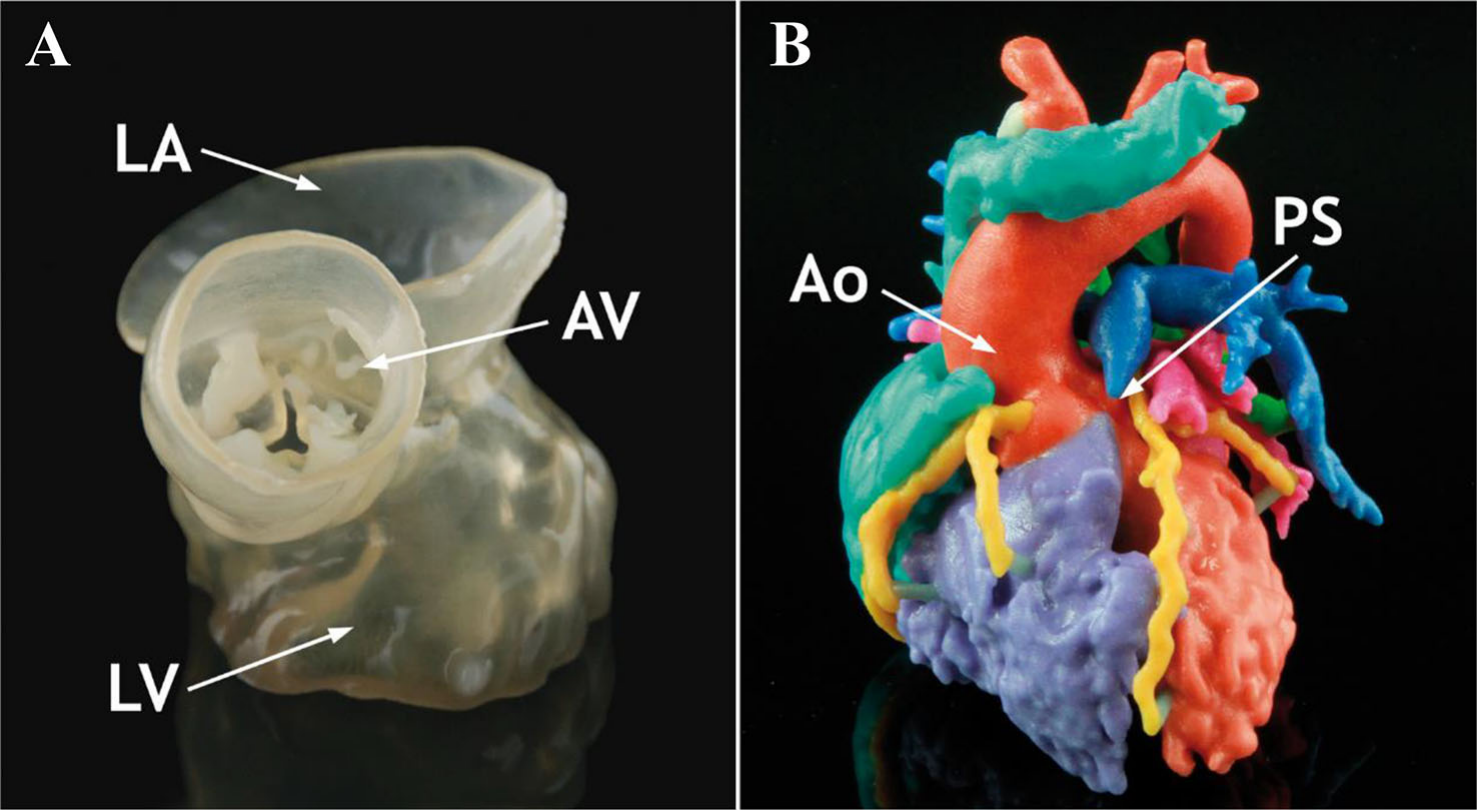
Anatomical 3D models of heart in normal and pathological state. **(A)** Normal anatomical 3D printed heart model. **(B)** Tetralogy of Fallot anatomical 3D printed heart model. Reproduced, with permission, from (Bartel et al., 2018).

**Figure 8:**
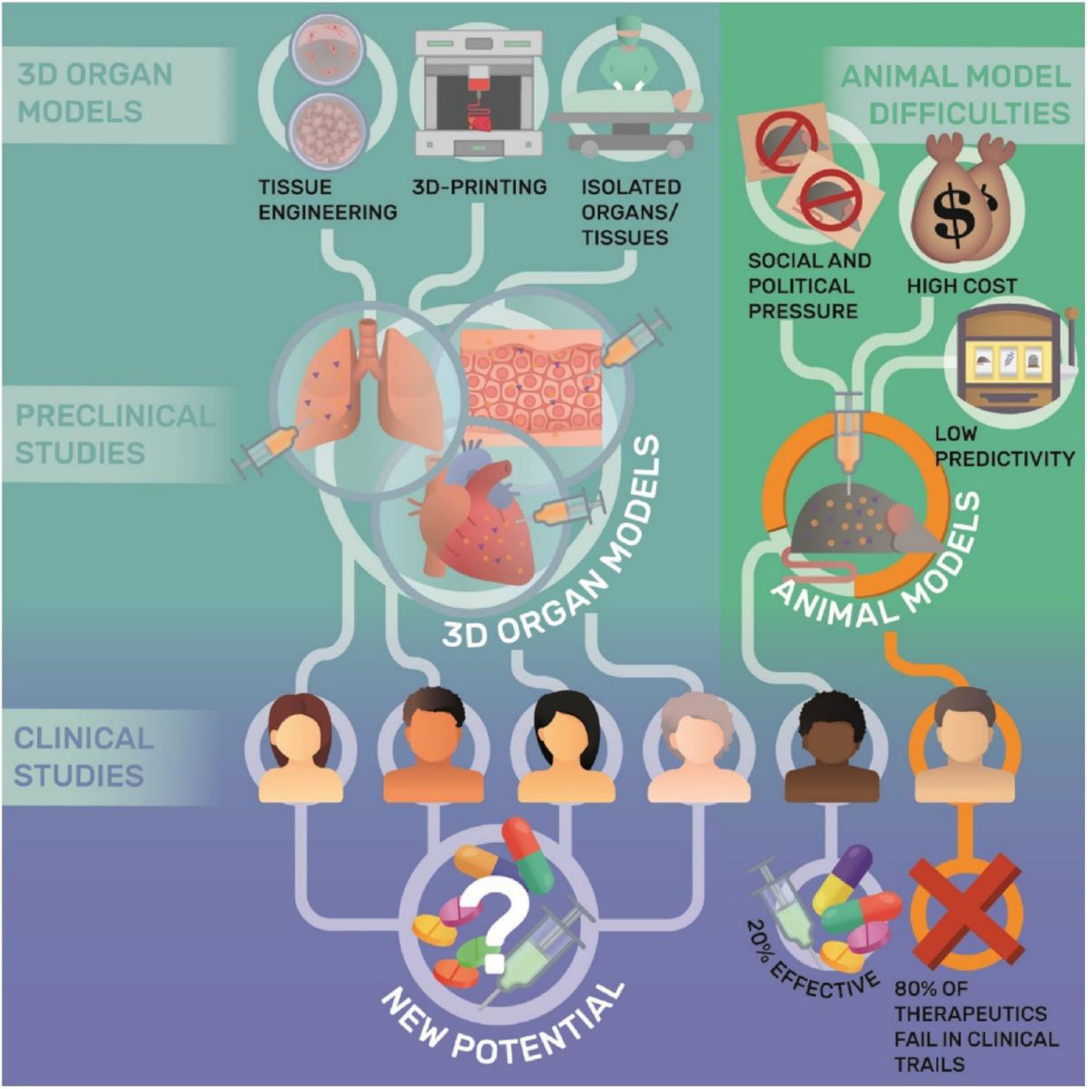
Applications and limitations of 3D organ models in pharmacological research. Reproduced, with permission, from (Weinhart et al., 2019).

To date, 3D printed tissue models play a significant role in the studies of mechanism of disease, pharmacological testing for new drugs, effectiveness of preclinical therapy, and anatomical structures of complicated organs (**Figures 7** and **8**). For these studies, conventional methods take plenty of mice and other experiment animals for building animal models. Typically, patient derived xenograft (PDX) models for medical studies always cost a large amount of immunodeficient mice to engraft disease cells. This kind of process takes a great mass of time and money. To overcome the disadvantage, tissue models were developed, firstly by traditional fabrication technologies without 3D printing. However, products by traditional methods revealed inaccurate models with unrealistic tissue status. With the application of 3D printing, biomimetic tissue models with high resolution are fabricated more efficiently at a lower cost than in the past. In this part, 3D printed tissue models of skin, liver, and tumor would be discussed.

The liver is a complex organ with multiple functions which have biotransformation effects on many non-nutritive substances (such as various drugs, poisons, and certain metabolites) in the body. They are completely decomposed by metabolism or excreted in the original form. With highly sensitive to drug toxicity, liver tissue engineering models were developed to take drug screening and testing. Reproducing the complex structures with 3D printing technology is the basic step for mimicking hepatic functions. To date, multiple fabrications of liver tissue models were accomplished with several 3D printing technologies. Ho-Joon Lee et al. developed multicellular 3D liver with multi-functions encapsulated in hybrid hydrogel (Lee et al., 2017). HepaRG cells alone or with supporting cells were encapsulated in semi-IPNs (hydrogel) and printed with vat polymerization technology. Fabricated 3D liver model was verified to be functional maturation with a dynamic 3D microenvironment, which is important for disease modeling and drug testing. Huanhuan Joyce Chen et al. fabricated a 3D scaffold co-cultured with human intestinal cells (hIECs) and liver cells to mimic a two-organ body-on-chip situation (Chen et al., 2018). The hIECs and liver cells in this scaffold were verified to maintain high viability and differentiable. While hIECs differentiated into human gastrointestinal cells, liver cells developed into lobule-like structures. Two organs on chip 3D model significantly improved the studies on human response and Inter-organ relationships. The two 3D liver models above were well fabricated and suitable for short-term studies. To realize long-term studies with functional liver tissue models, Hassan Rashidi et al. developed a stable 3D liver tissue model with certain function, which is testified for 1 year (Rashidi et al., 2018). Mimicking realistic conditions, hexagonal scaffolds were fabricated with polycaprolactone embraced with self‐aggregated pluripotent stem cells (PSCs) spheroids. Embedding with PSCs-loaded implants, two mice models of tyrosinemia were claimed to heal without any infection. Emerging 3D liver tissue models are helping solving problems in an efficient and cost-effective way that we cannot imagine before.

As one of the largest organs in human body, skin covers the whole-body surface and plays an important role in protecting, excreting, regulating body temperature, and feeling external stimuli. For patients with extensive skin wounds, clinical therapies would be complicated and important. To test the efficacy and safety of treatment, skin tissue models reveal an irreplaceable role. Byoung Soo Kim et al. fabricated a 3D printing skin tissue model with skin-derived extracellular matrix (S-dECM) bioink (Kim B. S. et al., 2018). Embraced *in vivo* with endothelial progenitor cells (EPCs) and adipose-derived stem cells (ASCs), 3D printing skin model accelerated wound healing especially in reepithelization and neovascularization. John W. Wills et al. adapted nanoparticles in 3D reconstructed skin micronucleus (RSMN) assay (Wills et al., 2016). After normalizing the dose between the total nanoparticle mass and the cell number between 2D/3D assays, the 3D dose response was compared to the 2D micronucleus assay. Due to the protective properties of the 3D cell microstructure and the mixed barrier effect, tested silica particles revealed no (gene) toxicity for live cells in the 3D model comparing to the 2D assay. Plenty results suggested 3D skin model can more accurately reflect the toxicity of nanoparticle drugs on human skin function than traditional methods.

Tumor is a new pathological organism formed by the proliferation of local tissue cells under the action of various tumorigenic factors, and has an extremely complex microenvironment and microstructure. It is significant to mimic *in vivo* tumor environment with stroma and micro structures for the accuracy of testing new theories and therapies (Costa et al., 2016). Jizhao Li et al. developed a 3D cell model with human lung cancer A549 cells applied in scaffolds fabricated with silk fibroin protein and chitosan (Li et al., 2018). By resembling pathological conditions, the 3D tumor model provide a valuable biomaterial platform for *in-vitro* test of antitumor drugs for non-small cell lung cancer. Yu Zhao et al. fabricated a 3D *in vitro* cervical tumor model with 3D printing of Hela cells in hydrogel grid structure by a layer-by-layer fashion (Zhao et al., 2014). With higher proliferation rate and matrix metalloproteinase protein expression, 3D cervical model fabricated with novel 3D printing technology is helping cervical tumor studies. Therefore, with the development of 3D printing tissue models, it is credible for the promising future that studies will be done more efficiently without sacrifices from experimental animals.

### Medical Apparatus and Instruments

**Figure 9:**
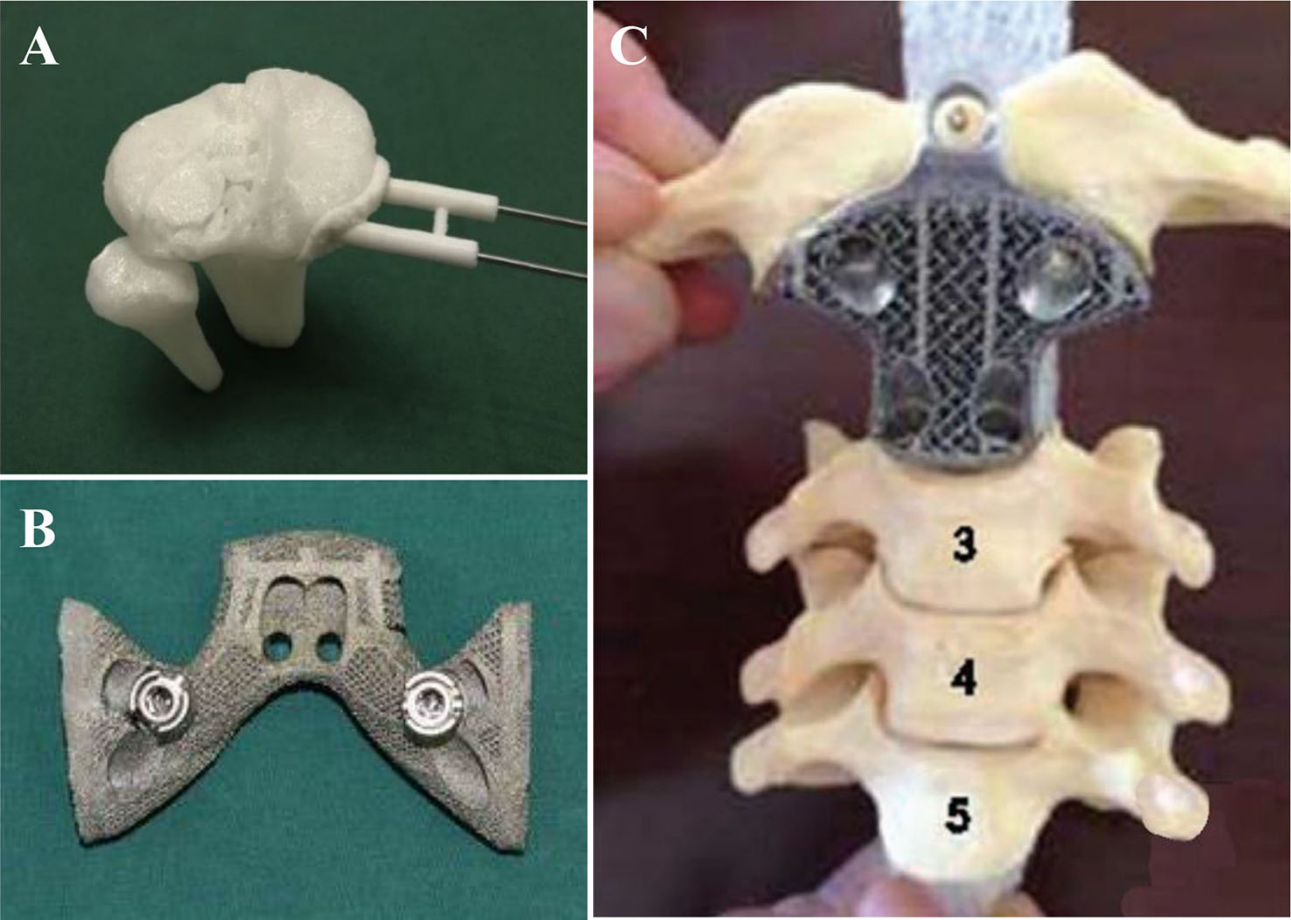
Medical apparatus and instruments by 3D printing. **(A)** 3D printed guide template for surgery simulation. **(B, C)** 3D printed titanium apparatus for cervical spine and pelvic surgery respectively. Reproduced, with permission, from (Xu et al., 2016; Wei et al., 2017; Nie et al., 2019).

AM is a promising and novel technology for the production of medical apparatus and instruments comparing with traditional manufacturing techniques. Directed by patient's clinical images, custom-designed medical apparatus and surgical guides are fabricated efficiently and accurately. It brings anatomically fit to patients and surgical safety to surgeons. Besides, AM is capable of manufacturing complex microstructures which are not possible for conventional techniques (**Figure 9**). With these advantages, AM allows fast production with high resolution, few leftover material and low costs. In this section, discussion is focused on (i) prostheses and implants and (Heidari Keshel et al., 2016) auxiliary medical equipment.

Medical implants and orthoses/prostheses (O&P) have been fabricated with traditional methods for decades. As with long term application, conservative implants revealed problems including anatomical mismatching, incompetent binding strength and initial stability, poor bone ingrowth and long-term stability, and low cost-efficiency (Wang et al., 2019). All these problems have been solved with AM technology, which is capable to fabricate implants with proper surface and mechanical properties. Powder-based 3D printing techniques (SLM, SLS, and EBM) are widely applied in implants and O&P manufacturing as they are compatible with a wide range of printing materials, such as titanium alloy, zinc alloy, cobalt–chrome alloy, and polyetheretherketone (PEEK). With outstanding mechanical properties and biocompatibility, 3D printed implants have been applied in plenty of surgical majors, including tracheobronchial (Zopf et al., 2014; Han et al., 2018), dentofacial, cardiovascular, orthopedic, and spine. For severe tracheobronchomalacia patients, a 3D printed self-expandable, metallic tracheobronchial stent fabricated with SLS technology was implanted into patient's collapse bronchus and rebuilt airway efficiently (Han et al., 2018). The printing technology offer a great opportunity of reconstruction and support for tracheobronchial diseases, which was difficult for conservative implants to be fabricated anatomically fitted. Besides self-expandable stent, a 3D printed bioresorbable stent was fabricated with SLS technique (Zopf et al., 2014). Printed bioresorbable stents were embedded into severe tracheobronchomalacia pig model, significant resolution of symptoms was observed. The stent was resorbed over time and was considered as a “4D” functional material. In maxillofacial and craniofacial surgeries, complex anatomy structures and irregular shapes of defects are the two most severe challenges. Conventionally, craniofacial prostheses are fabricated with hand-curved wax model for the anatomic defect with low precision. With 3D printing techniques, patient-customized prostheses are fabricated with guidance from CT or MRI images in which details for defects are well recorded. *Kyle K et al.* demonstrated the first application case of 3D printing in complex fetal craniofacial anomalies and perinatal management (VanKoevering et al., 2015). Researchers from Saint Louis University School of Medicine reviewed 315 patients who underwent 3D printing assisted maxillofacial and craniofacial surgeries (Jacobs and Lin, 2017). Fabrications with 3D printing techniques were mainly focused on contour models, surgical guides, splints, and implants. These objects were mainly fabricated in factory and laboratory with an average time and cost of 18.9 h and $1,353.31 respectively. Without lab or proficiency with printing software, low-cost 3D maxillofacial models could be fabricated with a cost of only $90 (Legocki et al., 2017). While commercial models can be manufactured with serializable materials and advanced virtual planning, this low-cost method can generate models with high-fidelity as educational and surgical planning tools. Cardiac diseases have been widely studied with the assistance of 3D printing technology as it offers high-resolution reduction of pathological status (Vukicevic et al., 2017). Variety of printing techniques including material jetting (Olivieri et al., 2015; Lind et al., 2017; Lau et al., 2018; Su et al., 2018), FDM (Mahmood et al., 2015; Son et al., 2015), SLS, and SLA have been applied in the studies of structural heart disease, congenital heart disease, coronary arteries, and systemic vasculature. Benefiting from 3D printing techniques, advanced visualization (Mahmood et al., 2015; Olivieri et al., 2015), diagnosis (Son et al., 2015), planning of surgeries, interventions (Lind et al., 2017), education (Lau et al., 2018; Su et al., 2018), and researches (Mahmood et al., 2015; Lind et al., 2017) in cardiovascular diseases are developing rapidly.

## Conclusion

This paper reviews the advancements of 3D printing technologies applied in medical materials in recent years. With the superiority of patient-specific designs, high complexity, favorable productivity, and cost-effective manufacturing methods, 3D printing has been becoming widely accepted manufacturing technologies in the medical applications. The main applications of 3D printing in medicine include tissue engineering models, anatomical models, pharmacological designs and validation models, medical apparatus and instruments. Orthopedics is one of the most advanced fields that integrate 3D printing to produce end-use products such as restorations, spine models, and surgical navigation boards. Orthopedics is a pioneer in medical devices. Currently, there are many multiple 3D printed medical products on the market, including implantable craniofacial implants, acetabular cups, knee implants, spinal cages, and surgical instruments. In addition, about 99% of hearing aid housings are custom made through 3D printing. Pre-surgery printed anatomical models have revolutionized the way surgeons and medical students were trained for surgery. To date, researchers have printed about 16 different types of tissues, providing tissue models for high-throughput screening for new drugs. It is believed that 3D printing is affecting clinical and related basic research in an increasingly broader manner.

## Author Contributions

DF and YL contributed equally to this reviewed manuscript. XW, YT and ZL conceived and designed the content of the manuscript. DF, YL and XW collected the researched literatures, arranged the outline of collected documents and wrote the articles. TZ, QW, HC, and WL made important suggestions and helped revising the manuscript. All authors reviewed and commented on the entire manuscript.

## Funding

This research was funded by the Ministry of Science and Technology of China (2016YFB1101501 and 2018YFE0104200) and National Natural Science Foundation of China (NSFC, 51973226).

## Conflict of Interest

The authors declare that the research was conducted in the absence of any commercial or financial relationships that could be construed as a potential conflict of interest.

The handling editor and reviewer JD declared their involvement as co-editors in the Research Topic, and confirm the absence of any other collaboration.
